# Correction

**DOI:** 10.1080/20018525.2025.2580853

**Published:** 2025-11-06

**Article title**: Airway hyperresponsiveness to mannitol in relation to inspiratory and expiratory resistance in subjects with asthma, COPD, and healthy smokers

**Authors**: Abir Nasr, Georgia Papapostolou, Linnea Jarenbäck, Kerstin Romberg, Alf Tunsäter, Jaro Ankerst, Leif Bjermer and Ellen Tufvesson

**Journal**: *European Clinical Respiratory Journal*

**Bibliometrics**: Volume 12, Number 01, pages 1-12

**DOI**: https://doi.org/10.1080/20018525.2025.2546677

It has been noted by the authors that Tables 1 and 2 were not included in the originally published article. The correct Tables 1 and 2 are provided below, and the original article has been updated to include them.

**Table 1.** Subject characteristics.

	Asthma	Healthynever-smokers		COPD	Healthy smoker		
	n=239	n=15	p-value^e^	n=25	n=14	p-value^f^	p-value^g^
Age, years	49(34–58)	47(34–66)	0.62	69(66–74)	60(58–67)	0.0043	<0.0001
Sex, females/males,n (%)	127/112 (53/47)	8/7(53/47)	0.98	20/5 (80/20)	8/6(57/43)	0.13	0.01
Height, m	1.73(1.67–1.80)	1.74(1.69–1.81)	0.86	1.65(1.61–1.72)	1.70(1.67–1.79)	0.021	0.0002
BMI, kg/m^2^	25 (23–28)	23 (22–25)	0.12	26 (24–29)	26(22–31)	0.57	0.18
FeNO^a^, ppb	19 (12-31)	20 (15-24)	0.86	13(9-18)	8 (6–12)	0.04	0.0027
Atopy^b^, n (%)	148 (62)	n.d.	n.a.	1 (4)	0	>0.99	<0.0001
Eosinophil^c^	0.2 (0.10–0.3)	0.1(0.1-0.15)	0.019	0.2 (0.2–0.4)	0.1(0.1–0.2)	0.0088	0.24
Non-smoker/ex-smoker^d^/smoker, n	157/8/74	15/0/0	0.021	1/17/7	0/2/12	0.001	<0.001
Packyears	0(0–1)	n. a.	n.a	26(13–39)	38(30–48)	0.061	<0.0001

Data is presented as median (IQR) if not otherwise stated. ^a^FeNO was missing in 10 subjects (5 asthma, 1 healthy never-smoker, 3 COPD and 1 healthy smoker).^b^One missing phadiatop test (atopy) in COPD.^c^Eosinophil test was not performed in 8 subjects (1 asthma and 7 healthy never-smokers).^d^Ex-smokers were defined as former smokers with ≥ 5 pack-years of smoking history. ^e^Comparison between asthma and healthy never-smokers.^f^Comparison between COPD and healthy smokers. ^g^Comparison between asthma and COPD. n. a.=non-applicable, BMI=body mass index, FeNO=fractional exhaled NO.

**Table 2.** Correlations between the relative change in % from baseline to last dose in FEV_1_ (=Δ%FEV_1_) and in respiratory oscillometry indices (Δ%R5, Δ%R19 and Δ%X5) among subjects with negative and positive mannitol test.

		Δ%R5_insp_	Δ%R5_exp_	Δ%R19_insp_	Δ%R19_exp_	Δ%X5_insp_	Δ%X5_exp_
**Asthma**							
Neg. mannitol test	**r**	0.049	0.12	0.031	0.020	0.019	0.11
	**p**	0.54	0.13	0.70	0.80	0.82	0.18
Pos. mannitol test	**r**	0.013	-0.016	0.10	-0.044	-0.12	-0.044
	**p**	0.91	0.89	0.35	0.69	0.28	0.69
**COPD**							
Neg. mannitol test	**r**	0.35	0.23	0.22	-0.28	**0.67**	0.27
	**p**	0.27	0.47	0.50	0.38	**0.02**	0.39
Pos. mannitol test	**r**	0.028	0.46	0.17	0.24	0.38	0.44
	**p**	0.93	0.11	0.57	0.42	0.19	0.13
**Healthy Smokers**							
Neg. mannitol test	**r**	0.57	0.11	-0.14	-0.25	0.32	-0.50
	**p**	0.20	0.84	0.78	0.59	0.50	0.27
Pos. mannitol test	**r**	-0.36	**-0.82**	*-0.75*	**-0.82**	-0.64	-0.32
	**p**	0.44	**0.034**	*0.066*	**0.034**	0.14	0.50

r=Spearman´s rank correlation coefficient.

This correction does not affect the description, interpretation, or conclusions of the article.

